# Immunological Mechanisms Underlying Chronic Pelvic Pain and Prostate Inflammation in Chronic Pelvic Pain Syndrome

**DOI:** 10.3389/fimmu.2017.00898

**Published:** 2017-07-31

**Authors:** María L. Breser, Florencia C. Salazar, Viginia E. Rivero, Rubén D. Motrich

**Affiliations:** ^1^Centro de Investigaciones en Bioquímica Clínica e Inmunología (CIBICI-CONICET), Departamento de Bioquímica Clínica, Facultad de Ciencias Químicas, Universidad Nacional de Córdoba, Córdoba, Argentina

**Keywords:** chronic prostatitis/chronic pelvic pain syndrome, prostatitis, inflammation, pelvic pain, autoimmunity, mast cells, Th1, Th17

## Abstract

Chronic prostatitis/chronic pelvic pain syndrome (CP/CPPS) is the most common urologic morbidity in men younger than 50 years and is characterized by a diverse range of pain and inflammatory symptoms, both in type and severity, that involve the region of the pelvis, perineum, scrotum, rectum, testes, penis, and lower back. In most patients, pain is accompanied by inflammation in the absence of an invading infectious agent. Since CP/CPPS etiology is still not well established, available therapeutic options for patients are far from satisfactory for either physicians or patients. During the past two decades, chronic inflammation has been deeply explored as the cause of CP/CPPS. In this review article, we summarize the current knowledge regarding immunological mechanisms underlying chronic pelvic pain and prostate inflammation in CP/CPPS. Cumulative evidence obtained from both human disease and animal models indicate that several factors may trigger chronic inflammation in the form of autoimmunity against prostate, fostering chronic prostate recruitment of Th1 cells, and different other leukocytes, including mast cells, which might be the main actors in the consequent development of chronic pelvic pain. Thus, the local inflammatory milieu and the secretion of inflammatory mediators may induce neural sensitization leading to chronic pelvic pain development. Although scientific advances are encouraging, additional studies are urgently needed to establish the relationship between prostatitis development, mast cell recruitment to the prostate, and the precise mechanisms by which they would induce pelvic pain.

## Chronic Pelvic Pain

Chronic pelvic pain is generally defined by chronic pain in the region of the pelvis (1). It is a common symptom of several structural and functional disorders affecting the anorectal area, urinary bladder, reproductive system, and pelvic floor musculature and its innervation (2). In contrast to structural diseases such as endometriosis, the pelvic pain in functional disorders cannot be explained by an organic or other specified morphological pathology (3). Functional disorders are classified into anorectal (e.g., proctalgia fugax, levator anisyndrome, and unspecified anorectal pain), bladder [e.g., interstitial cystitis (IC)/bladder pain syndrome], and prostate syndromes [e.g., chronic prostatitis/chronic pelvic pain syndrome (CP/CPPS)]. IC/bladder pain syndrome is primarily diagnosed in women, whereas CP/CPPS is a diagnosis exclusive to men. Although IC and CP/CPPS have been largely considered different, they share many clinical features and are currently classified under the umbrella term, *urologic chronic pelvic pain syndromes* (4).

Chronic pelvic pain experienced by patients bearing CP/CPPS presents as chronic pain (that lasts for at least 3–6 months) in the region of the pelvis, perineum, scrotum, rectum, testes, penis, and often associated to ejaculatory pain, pain in lower back and abdomen, often associated to lower urinary tract symptoms, erectile dysfunction, and psychosocial symptoms. Lower urinary tract symptoms may include obstructive and/or irritative voiding symptoms. Erectile dysfunction is a major concern for CP/CPPS patients, which is defined as the persistent inability to attain and maintain a penile erection that is sufficient for satisfactory sexual performance (5). All these complaints lead to patient frustration, diminished quality of life as well as impairments in primary intimate relationships. Moreover, there is a common association of CP/CPPS with other systemic syndromes such as irritable bowel syndrome, fibromyalgia, cardiovascular disease, stress, depression, and anxiety (6). In fact, in terms of pain and deteriorated quality of life, CP/CPPS patients have shown to have a quality of life comparable with that of patients who have suffered myocardial infarction or bear Crohn disease (7).

## Chronic Prostatitis/Chronic Pelvic Pain Syndrome

The term prostatitis defines as a state of inflammation of the prostate gland. The currently used classification of prostatitis was proposed by the National Institutes of Health (NIH) in 1999 (8). Prostatitis syndromes are divided in four categories: acute and chronic bacterial prostatitis (types I and II), CP/CPPS (type III), and asymptomatic inflammatory prostatitis (type IV). Acute and chronic bacterial prostatitis are characterized by uropathogenic infections in which causative pathogens can be detected in the semen, in expressed prostate secretions (EPS), or urine after prostatic massage; and these infections respond well to antibiotic therapy (9, 10). In contrast, CP/CPPS or NIH type III prostatitis is a complex and frustrating disease for both, patients and physicians, with symptoms that are difficult to quantify as well as to effectively treat. CP/CPPS is defined by chronic pelvic pain and signs and symptoms of prostate inflammation, lasting for at least 3–6 months, in the absence of any detectable infection. These features distinguish it from the other types of prostatitis (11, 12). CP/CPPS presents with a mixture of chronic pelvic pain, lower urinary tract symptoms, and ejaculatory/sexual complaints with no uniformly effective therapy (6, 13). Noteworthy, CP/CPPS is one of the most common diseases frequently diagnosed in the fields of urology and andrology (10). Moreover, it has been revealed that CP/CPPS may have significant consequences in male fertility (10, 14). It accounts for more than 90% of all cases of prostatitis diagnosed and it has been estimated that affects 9–16% of men of all ethnic origins and is the most common urologic morbidity in men younger than 50 years old (15–17).

Chronic prostatitis/chronic pelvic pain syndrome is a syndrome, thus patients may be very heterogeneous and present a widely variable array of symptoms. In response to that, two multimodal approach systems are currently used to assess CP/CPPS symptom severity and to help physicians to manage patients: the CP Symptom Index from the NIH (NIH-CPSI) and the urinary, psychosocial, organ specificity, infection, neurologic, and tenderness (UPOINT) system (18, 19). On the one hand, the NIH-CPSI intends to assess symptoms severity and to quantify their impact on the patients’ quality of life. However, it should be noted that is generally based on a subjective questionnaire and overall scores are determined by a cumulative scoring of symptoms that might and might not be related to one another, or indeed to the underlying causes of pathology. The NIH-CPSI is a validated nine question survey that covers the following three domains: pain (location, frequency, and severity), urinary symptoms, and quality of life (18). Using this system, CP/CPPS is diagnosed when patients present with pelvic pain and an index score higher than 4. A six-point improvement in total score is considered clinically significant correlates with patient reported improvement (20). On the other hand, the UPOINT system can be used to identify clinical phenotypes and can also be used to direct therapy. This system considers each group of patients’ symptoms and divides them into six categories: urinary, psychosocial, organ specificity symptoms, infection, neurologic/systemic, and tenderness. The characteristics and advantages of this classification system and its implications in therapy prescription were excellently analyzed in a recent review by Polackwich and Shoskes (6). As a reflex of the diversity of patients’ symptoms, the median number of positive UPOINT domains is 3 and only 22% of patients have a single positive domain. In addition, UPOINT domains involved have been shown to correlate with NIH-CPSI scores (21, 22), and also help in guiding the practitioner to prescribe therapy. The use of this system treatment strategy is starting to become more widespread and is proving its effectiveness significantly improving the patients’ quality of life (23, 24).

## Possible Causes of CP/CPPS

Available therapeutic options for CP/CPPS are far from satisfactory for either physicians or patients. A novel, effective therapeutic approach is urgently needed (6, 25, 26). The main reason for the lack of effective and uniform therapies is that the etiology of CP/CPPS still remains unknown (27). Most likely, CP/CPPS comprises similar clinical phenotypes resultant from a combination of different pathophysiological mechanisms. During the last two decades, research in the field has experienced a notable growth. Several hypotheses have been proposed to explain CP/CPPS pathogenesis including defective urothelial integrity and function, cryptic infections, autoimmunity, endocrine imbalances, pelvic floor muscle spasm or tenderness, voiding dysfunction, peripheral and central sensitization and neuroplasticity, and psychosocial conditions (1, 2).

### Infection

Infection has been historically assumed to be the cause of CP/CPPS; thus, it has been empirically treated with antibiotics although with limited success. In this regard, several studies have systematically failed to identify infectious agents as causative agents of this pathology (28). Moreover, most patients’ symptoms are refractory to antibiotic therapy (6). In a recent study and using molecular techniques, Nickel et al. found that the overall species and genus composition differed only in the initial urine stream of CP/CPPS patients versus controls, being *Burkholderia cenocepacia* overexpressed in CP/CPPS patients. In contrast, midstream or postprostatic massage samples were not significantly different (29). Although the presence of an active infection in patients was not evident in almost all studies carried out up to date, CP/CPPS patients were found to have a significantly greater history of urethritis compared with age-matched controls (30). While a primary infectious agent may not be the cause of the ongoing symptoms, infection may be the precipitating factor. In this regard, different microorganisms have been implicated such as *Chlamydia trachomatis, Mycoplasma hominis, Ureaplasma urealyticum, Trichomonas vaginalis, Candida* spp., herpes simplex virus, etc. (26). In susceptible men, infectious urethritis or prostatitis could serve as the initial stimulus for chronic inflammation, although chronic inflammation and pain may persist after the infection has been cleared, possibly by an autoimmune, and/or neurogenic mechanism. If this is the case, infection would be the triggering factor rather than the cause of the pathology. In fact, using an animal model of prostatitis, the uropathogenic CP1 strain of *Escherichia coli*, isolated from a patient with CP/CPPS, has been shown to induce and sustain chronic pelvic pain that persisted long after bacterial clearance from the mouse genitourinary tract. Pelvic pain was produced in the NOD strain of mice but not in C57BL/6 mice despite similar invasion and proliferation in each species. This indicates a genetic susceptibility to chronic inflammation and pain but not to the infection itself (31). The NOD mice that developed pain are genetically prone to develop chronic inflammatory conditions in different organs (32). As stated above, infections may act as triggering factors. Consequently, patients may develop inflammation and/or neuronal damage confined to the prostatic or pelvic area, which may be further augmented by the localized chronic inflammatory milieu. The unresolved chronic inflammation may potentiate tissue injury leading to pelvic floor dysfunction (33) and central sensitization resulting in chronic pelvic pain (34).

### Pelvic Floor Dysfunction

Pelvic floor dysfunction as increased pelvic floor muscle spasm or tenderness has been also proposed as responsible for CP/CPPS symptoms. In fact, spasms or tight knots or trigger points in the pelvic floor muscles lateral and anterior to the prostate have been shown in CP/CPPS patients. It is recommended that practitioners should perform careful palpation of these muscles during the rectal exam in order to reproduce the patient’s primary pain and to distinguish pain due to spasm from pain consequence from inflammation or other conditions (6). Besides, assessment of chronic pelvic pain tenderness by ultrasonography has linked pelvic floor muscle spasm to CP/CPPS (35). However, whether pelvic floor muscle spasm is the causative agent or direct mediator of CP/CPPS symptoms still remains to be established.

### Chronic Inflammation/Autoimmunity

Chronic inflammation has been deeply explored as the cause of CP/CPPS during the past two decades. Cumulative evidence points to the possibility that this syndrome is a consequence of dysregulated inflammation in the form of autoimmunity directed against prostate antigens (PAg). Indeed, an autoimmune basis for CP/CPPS is a very prominent theory based on substantial evidence from studies in patients and animal models (Table 1) (10, 36–38).

**Table 1:** Immune findings in patients and animal models (experimental autoimmune prostatitis) of chronic prostatitis/chronic pelvic pain syndrome (CP/CPPS).

Main finding	Reference
**Findings in CP/CPPS patients**	
Specific T cell responses and IFNγ secretion to prostate antigens (PAg) and seminal proteins	(39–44)
Serum PAg-specific IgG	(45)
Prostate tissue Ig deposition, prostate leukocyte, and T cell infiltration	(46–48)
Increased numbers of leukocytes (granulocytes, macrophages, T, and B cells) in expressed prostate secretions (EPS), urine after prostatic massage, or semen	(16, 29, 49–51)
Increased levels of immunoglobulins, inflammatory cytokines, chemokines, and mast cell mediators in EPS or seminal plasma	(16, 47, 50, 52–60)
**Findings in animal models of autoimmune prostatitis**	
Macrophages, DCs, mast cells, CD4+ and CD8+ T and B cells infiltrating the prostate	(61–72, 79–91)
CD4+ T cells are essential in driving prostatitis	(63)
Th1/Th17-associated autoimmune responses to PAg	(64–66, 83, 91)
PAg-specific immune response is associated to a Th1 cytokine and immunoglobulin isotype pattern	(83, 84)
Crucial role of IFNg in mediating pathology	(62, 65–67)
CXCR3 and CCR5 expressing PAg-specific Th1 cells mediate disease induction	(66)
IL-17 is dispensable for disease and pain development	(67)
Treg function condition disease and pain induction	(68)
Pelvic pain development correlated with inflammation	(67, 69)
Increased tryptase-B and nerve growth factor (NGF) in prostate tissue	(70)
Mast cells mediate pelvic pain development	(71)
CCL2, CCL3, and tryptase-B involved in pain development	(72)
Increased NGF and neuronal density in prostate tissue	(73)

#### Evidence from Studies in Patients

Evidence from studies in patients is summarized in Table 1. Self-reactivity of T cells from CP/CPPS patients to PSA, prostatic acid phosphatase, and other prostatic and seminal plasma proteins has been reported in several human studies (39–44). In fact, the presence of IFNγ-secreting Th1 lymphocytes specific to PAg and peptides has been reported in an important fraction of CP/CPPS patients (42–44). In addition, increased serum levels of IgG specific to the PAg MAD-PRO-34 and Ny-Co-7 have been detected in CP/CPPS patients versus controls (45). Also, IgM and IgA antibody deposition with no infectious specificity has been shown in prostate tissue samples from CP/CPPS patients (46). Moreover, the presence of prostatic intra-acinar T cell rich infiltrates has been described in CP/CPPS patients (47, 48). The analysis of EPS from men with CP/CPPS showed increased numbers of leukocytes (16, 29, 49–51). Characterization of these infiltrates in ejaculates as well as in prostate tissue samples revealed increased numbers of granulocytes, macrophages, and activated T and B lymphocytes. In addition, high levels of inflammatory cytokines, chemokines, and mast cell degranulation products have been demonstrated in clinical samples from CP/CPPS patients suggesting an active inflammatory process of the male genital tract in the absence of infection (10, 12, 60). Elevated levels of IL-1β, TNFα, IFNγ, IL-6, IL8, MCP-1/CCL2, MIP-1α/CCL3 as well as decreased levels of IL2R (16, 47, 50, 52–60) have been shown in seminal plasma, EPS, and/or urine after prostatic massage from CP/CPPS patients. IL8 has been proposed as a valuable biomarker since its levels strongly correlated to CP/CPPS severity; patients that showed higher IL8 levels presented worse symptoms (58, 74). Prostate biopsies revealed inflammation in 33% of patients with CP/CPPS (75). Remarkably, IFNγ-producing Th1 cells specific to PAg and antigen recognition restriction to certain HLA-II haplotypes were detected in CP/CPPS patients (39, 42–44, 76), suggesting a Th1 autoimmune response against the prostate as the underlying disease mechanism (10). Since Th17 cells have been implicated in the pathogenesis of different autoimmune diseases (77), some authors have speculated that IL-17 would be involved in CP/CPPS, particularly mediating chronic pelvic pain development (38). However, they failed in identifying IL-17 in clinical samples from CP/CPPS patients (64) and to the best of our knowledge, no reports in humans have been published about the role of IL-17 produced by Th17 cells in CP/CPPS.

#### Evidence from Animal Models

Different animal model have been developed for the study of CP/CPPS (36, 37, 78). Animal models, by definition, cannot perfectly reflect human disease; yet medical history is replete with mechanistic insights gleaned from animal studies that were otherwise impossible, too invasive, or unethical to obtain from human studies. Cumulative evidence for an autoimmune basis for CP/CPPS comes from animal models of experimental autoimmune prostatitis (EAP) that have proven to be reliable for the study of CP/CPPS and have provided important data about the immune mechanisms underlying disease induction, development, and pathological consequences (Table 1). EAP, non-infectious autoimmune animal models of CP/CPPS achieved by immunization of rats or mice with PAg plus adjuvants, have been studied since several decades ago (61, 79–82). EAP models mirror the human disease showing its typical characteristics: the presence of IFNγ-secreting Th1 lymphocytes specific to PAg, increased levels of cytokines in semen, chronic pelvic pain, and associated prostate tissue inflammation and lesions (10, 36, 37, 72, 83). Immunization with prostate gland homogenates (83–86), or purified prostate proteins such as prostate steroid binding protein and prostate acid phosphatase (63, 83, 87, 88) or peptides (62, 89), induces PAg-specific Th1 cell and antibody responses associated with histological evidence of prostate inflammation and the induction of chronic pelvic pain (62, 66, 67, 83, 89, 90). Moreover, IFNγ or transcription factors involved in IFNγ signaling, such as IRF1 and STAT1, have been shown to be crucial in EAP induction, supporting the Th1 nature of the immunopathogenic underlying mechanism (62, 65, 66). Also, the expression of the Th1-associated chemokine receptors CXCR3 and CCR5 on prostate-specific pathogenic T cells was shown to be associated with their homing and infiltration of the prostate (66). Some authors have argued for a role of Th17 cells in EAP induction and, especially, for IL-17 in the development of chronic pelvic pain (38, 62, 64, 91, 92). They found elevated levels of IFNγ and IL-17 in the inflamed prostate tissue of mice with prostatitis, and also *ex vivo* when prostate-draining lymph node T cells from these mice were *in vitro* stimulated (62, 64, 91). Nevertheless, it has been shown that the immunization of BALB/c mice, a strain resistant to EAP induction, also induces elevated peripheral prostate-specific IL-17-secreting T cell responses, although these T cells are unable to infiltrate the prostate and cause the disease (66). In addition, although PAg immunized NOD-IFNγ-KO mice induce markedly increased peripheral frequencies of prostate-specific Th17 cells, they are resistant to EAP induction and pathology development (66). Interestingly, using NOD mice infected with the uropathogenic CP1 strain of *E. coli*, Quick et al. showed that the infection elicited a Th1/Th17 chronic inflammatory response that infiltrated the prostate and mediated chronic pelvic pain. These authors proposed that IL-17 produced by Th17 cells would be the main inducer of pain (92). However and as mentioned before, the autoimmune nature of this animal model and the exact role of IL-17 in mediating inflammation and pain were not proven (31, 92). In order to shed light on this controversial issue, we recently analyzed the precise role of different Th cell subsets in EAP pathogenesis and chronic pelvic pain development using IL-12p40-KO mice (Th1 and Th17 cell deficient), IL-4-KO mice (Th2 cell deficient), IL-17A/F-double KO mice (IL-17A/F deficient), and wild type (C57BL/6) mice (67). Our results demonstrated that PAg-specific Th1 cells induce prostate tissue inflammation and, in turn, provoke chronic pelvic pain, the hallmark symptom of CP/CPPS. Moreover, the absence of Th1 or Th2 cytokines, respectively, diminished or enhanced EAP susceptibility. However and most importantly, IL-17A or IL-17F production by Th17 cells has shown to be dispensable for immunopathology and pain development (67). Finally, additional evidence that highlights the importance of the genetic background in conferring differences in naturally operating regulatory T cell control mechanisms of inflammation in EAP and chronic pelvic pain development has been recently reported (68, 69). All the evidence obtained using EAP models reflect multiple and key features of human disease and help improving the understanding of the pathophysiological immune mechanisms that may underlie CP/CPPS.

Over the past decade, other animal models of CP/CPPS that induce chronic non-infectious inflammatory prostatitis have also been studied. They include the above-mentioned chronic inflammatory prostatitis model induced by the uropathogenic CP1 strain of *E. coli* (92), mechanical prostatitis (94), or chemical prostatitis induced by capsaicin (95, 96), formalin (97, 98), or complete Freund’s adjuvant (CFA) (99). Mechanical prostatitis induced by partial obstruction of the urethra from Wistar rats caused prostate lymphocytic infiltration and interstitial edema demonstrating that urinary reflux may be an etiologic factor in CP/CPPS (94). Intraprostatic injection of capsaicin, an agent thought to excite C-afferent fibers and cause neurogenic inflammation, induced chronic prostate inflammation and pelvic pain in a dose-dependent manner (95). Chronic inflammation evidenced by prostate tissue edema, augmented lymphocyte infiltration, and increased expression of COX-2 was accompanied by the induction of pelvic pain and increased COX-2 expression in spinal sensory and motor neurons, which were attenuated by intraprostatic instillation with botulinum toxin A (96). Similarly, chemical irritation of the prostate by intraprostatic injection of formalin induced inflammation, and plasma extravasation and increased expression of c-Fos and substance P (SP) within the lumbosacral spinal cord, which suggested that referred pain status in inflammation of the prostate is neurogenically mediated (97, 98). Finally, it was recently reported that injection of CFA into the prostate from Sprague–Dawley rats caused chronic prostate inflammation and pelvic pain (99). Prostate inflammation, shown by increased inflammatory cell infiltration and COX-2 expression levels, was accompanied with the induction of chronic pelvic pain, evidenced by behavioral changes, and increased expression of glial fibrillary acidic protein (GFAP) in the spinal cord. Moreover, treatment with melittin significantly alleviated pain by decreasing inflammatory infiltrates, and suppressing prostate COX-2 and spinal cord GFAP expression (99).

Altogether, these data support the notion that local chronic inflammation may induce pelvic pain through a neurogenic mechanism. Based on that, there should be significant overlaps of nociceptive neurons within the spinal cord, which receive nociceptive inputs from pelvic soma and viscera.

## Inflammation and Pain Development in CP/CPPS

One of the main and currently unanswered questions in CP/CPPS is how chronic pelvic pain develops and whether a mechanistic link with inflammation exists. Due to the unknown pathophysiological pain mechanisms involved and to the diverse range of symptoms, both in type and severity, presented by patients, no standard, and completely effective therapeutic approach exists. Wide ranges of therapies for pain control have been tested, but most have shown heterogeneous efficiency, particularly in pain management (27).

As for any organ system, male pelvic pain occurs as a physiological alarm to withdraw from an injurious (infectious, irritative, inflammatory, or traumatic) condition to reduce tissue damage (100). However, chronic pain may start after tissue damage or inflammation and remain subsequent to tissue healing, becoming harmful, and detrimental to health (101). Chronic pain is commonly triggered by chronic peripheral inflammation and nerve injury. These results in the release of neurotransmitters, lipid mediators, fragments of the complement system, neuropathic factors, cytokines, and chemokines in both the central and peripheral nervous system (101). Both, inflammatory and neuropathic pain can cause peripheral and central sensitization that can lead to allodynia, hyperalgesia, and spontaneous pain. Several authors specialized in this area suggest that inflammatory/neuropathic pain may play a central role in CP/CPPS (17, 33, 100, 102). Chronic pelvic pain include a combination of spontaneous visceral and referred somatic pain characteristics (i.e., pelvic visceral and referred perineal pain), and also the involvement of central sensitization in the spinal cord and brain (103). CP/CPPS patients have shown specific patterns of functional and pain-related brain activation and anatomical reorganization, which correlated with clinical pain intensity (104, 105). These changes were evident in regional and global scales, suggesting an ongoing reorganization of brain circuitry similar to other chronic pain morbidities such as musculoskeletal and neuropathic pain, chronic low back pain, postherpetic neuralgia, complex regional pain syndrome, and knee osteoarthritis (106). These findings suggest that the chronic presence of pelvic pain leaves specific brain neural imprints that persist for years. Alternatively, some of these neural abnormalities may be predisposing factors for CP/CPPS. However, it is unclear if these central changes are the cause or consequences of disease progression.

Although thalamic and brain cortical levels may be involved in pelvic pain in CP/CPPS, most attention has focused on the dorsal horn of the spinal cord. Central sensitization is caused by chemical and anatomical changes leading to hyperexcitability in the dorsal horn cells from persistent afferent C fiber bombardment by painful stimuli (102). Chronic pain is induced and maintained by mediators released by immune cells (macrophages, lymphocytes, and mast cells), neurons and glial cells that trigger peripheral and central sensitization (107). It has been proposed that neurogenic processes, autoimmune injury, and mast cells may contribute to inflammation and trigger pain development in CP/CPPS in males (108). Inflammatory stimuli are known to induce SP, calcitonin gene-related peptide, and nerve growth factor (NGF) secretion from nerve terminals, resulting in plasma extravasation, edema, and hyperalgesia, commonly referred to as neurogenic inflammation (109). Interestingly, animal models of prostate inflammation and pain have shown that chemokines and cytokines are crucial in sustaining or amplifying inflammation induced by SP (110, 111). In this regard, mast cells have been suggested to play a central role (109). Increased number of mast cells and their secretion products has been associated with chronic inflammatory pain conditions such as rheumatoid arthritis, multiple sclerosis, IC, and inflammatory bowel disease (109, 112–117). They are currently suggested as the main mediator and effector cells in disease progression from initiation to breaking of tolerance, neuronal activation, and, eventually, sensitization (109, 118, 119). Mast cells are tissue-resident immune cells that promote the infiltration of inflammatory cells such as macrophages and lymphocytes into tissues, which when activated secrete cytokines that further activate mucosal mast cells, thus perpetuating the cycle of inflammation. Moreover, mast cells respond to SP and NGF secreted by neuronal terminals degranulating and releasing histamine, serotonin, cytokines, chemokines, prostaglandins, and neuropeptides such as brain-derived neurotrophic factor, neurotrophin-3, and more NGF and SP (120, 121). Mast cells are known to express NGF receptors (TrkA, B, C) on their cell membrane and, therefore, NGF binding might cause degranulation and cytokine and chemokine release, establishing a feedback mechanism that would promote rapidly occurring sensitization mechanisms (121). The augmented density of sensory nerve fibers observed in prostatitis (72, 73) and inflammation induced by mast cell activation and degranulation might result in irreversibly altered neurotransmission and thus explain, at least partly, the chronic nature of pain in CP/CPPS. It has been shown that CP/CPPS patients have elevated levels of mast cell attractant chemokines CCL2 (MCP-1) and CCL3 (MIP-1α) in EPS, which associated with clinical pain (59). Moreover, elevated levels of mast cell tryptase-β, carboxypeptidase A3, and NGF were also detected in EPS and urine from CP/CPPS patients, and those NGF levels directly correlated with pain severity (70, 71, 122). These results suggest that NGF and mast cells secretion products are potential mediators involved in pain sensitization mechanisms in CP/CPPS. In agreement, treatment with pentosan polysulfate, a stabilizer of mast cells was shown to ameliorate symptoms in CP/CPPS patients (123). Besides, tanezumab, a monoclonal antibody against NGF, was evaluated for CP/CPPS treatment. However, clinical trials showed no significant improvement compared with placebo suggesting that anti-NGF therapy is not sufficient by itself to reduce symptoms (124).

**Figure 1:**
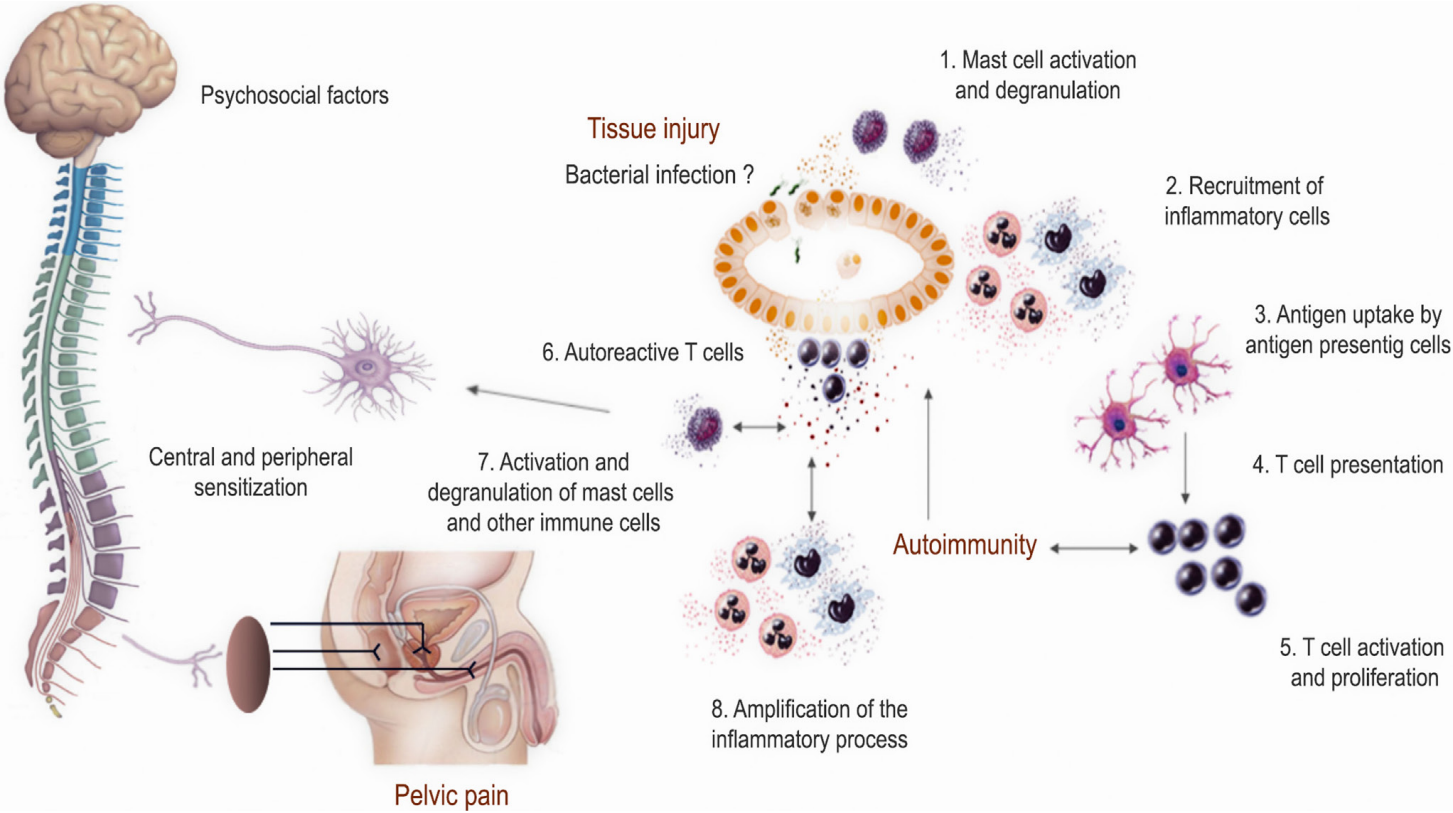
Proposed model of the pathophysiological mechanisms involved in prostate inflammation and chronic pelvic pain development in chronic prostatitis/chronic pelvic pain syndrome. This model resumes most evidence obtained from human studies and experimental animal models. Several factors may trigger chronic inflammation in the form of autoimmunity directed against prostate antigens and foster chronic prostate inflammation with the recruitment of different leukocytes including mast cells. The local inflammatory milieu and the secretion of inflammatory mediators may induce neural sensitization leading to chronic pelvic pain development.

The presence of markedly increased numbers of mast cells in prostate cell infiltrates was already reported in animal models of autoimmune prostatitis several years ago (125–129). Moreover, adoptive transfer of lymphocytes from animals with prostatitis to naïve recipients caused disease inducing prostate inflammation in terms of lymphocytic infiltration and the presence of mast cells (127) (Table 1). Furthermore, mast cells evidenced an activated state since most of them showed to be degranulated (21, 125–127, 129) and having secreted pain inducer molecules such as NGF and tryptase-β (70, 71). It has been shown that NGF sensitizes sympathetic neurons to proinflammatory stimuli (130). In fact, mice with EAP showed increased intraprostatic NGF levels, augmented neuronal density in prostate tissue, and also microglial activation in the spinal cord (71–73, 131). As observed in patients, increased levels of CCL2 and CCL3 were also demonstrated in EAP, which were suggested to play a major role in recruiting mast cells and mediating pain (60). In addition, mast cell-deficient Kit^W-sh^/Kit^W-sh^ mice have significantly decreased intraprostatic NGF levels and attenuated pain responses upon EAP induction (71). Cytokines secreted by mast cells have been shown to control Th17/Treg cell differentiation and plasticity (132). In this regard, Murphy et al. recently provided some controversial evidence indicating that IL-17 would be crucial for the induction but not maintenance of pelvic pain in EAP in C57BL/6 mice (64). Authors showed that prophylactic treatment with IL-17-blocking antibodies was sufficient to abolish pelvic pain development. However, they surprisingly did not show any data about prostate tissue inflammation or cell infiltration in order to definitively assess if pelvic pain was related to prostate inflammation. Besides, the authors remarkably showed that they failed in preventing or ameliorating chronic pelvic pain when administering a therapeutic treatment of IL-17-blocking antibodies on day 10 post EAP induction (64). In order to definitely assess the role of IL-17 in mediating chronic pelvic pain, we recently analyzed pelvic pain development and prostate inflammation induction in EAP using IL-17A/F-double KO and wild type (C57BL/6) mice (67). Our results demonstrated that wild type mice induced prostate-specific Th1 and Th17 immune responses that caused prostate tissue inflammation and chronic pelvic pain development, which augmented as disease progressed, suggesting that chronic pelvic pain development was a consequence of prostate inflammation. Interestingly, IL-17A/F-double KO mice induced a prostate-specific immune response, prostate tissue inflammation, and chronic pelvic pain development similar to wild type mice (67). Therefore, IL-17 was shown to be dispensable for prostate inflammation induction and chronic pelvic pain development. Furthermore, similar levels of mast cell infiltrates, in close contact to nerve fibers, were observed in IL-17A/F-double KO and wild type mice (unpublished data), in agreement with the similar pattern of chronic pelvic pain development and as previously reported (133). In summary, development of chronic pelvic pain was only shown in those animals that induced prostate-specific Th1 immune responses and subsequently prostate tissue inflammation, cell infiltration, and mast cell recruitment. In fact, PAg-specific Th1 cells express associated chemokine receptors such as CXCR3 and CCR5 and migrate to and infiltrate the prostate gland (66). Once there, these lymphocytes induce the local secretion of several cytokines and chemokines, including the ligands for CXCR3 and CCR5, which in turn recruit more leukocytes augmenting tissue cell infiltration and enhancing prostate inflammation and chronic pelvic pain development (62, 65, 66, 68, 69). Altogether, these evidence support the notion that within the Th1 induced leukocyte infiltrates, mast cells might be key actors in the consequent development of chronic pelvic pain (Figure 1). However, it remains to be established if removal of inflammation can reverse or ameliorate mast cell infiltration, neuronal sensitization, and chronic pelvic pain. Additional studies are needed to establish the relationship between prostatitis induction, prostate mast cell activation/degranulation, and the precise mechanisms by which they would induce chronic pelvic pain.

## Concluding Remarks

Chronic prostatitis/chronic pelvic pain syndrome is a complex and frustrating syndrome because of the unknown underlying pathophysiological mechanisms and the lack of appropriate and effective therapies. The emergence of interest and new research on the field has allowed a significant improvement in the understanding of this syndrome. Data obtained from studies in patients and animal models have confirmed the involvement of immune mechanisms in the etiology, pathogenesis, and chronic pelvic pain development. Several factors may trigger chronic inflammation in the form of autoimmunity directed against PAg and foster chronic prostate inflammation with the recruitment of different leukocytes including mast cells. The local inflammatory milieu and the secretion of inflammatory mediators may induce neural sensitization leading to chronic pelvic pain development (Figure 1). Further study of the available experimental animal models together with more extensive research in the human syndrome will shed light on the precise physiopathology of CP/CPPS, something that in turn may help in finding more rational and effective therapies.

## Author Contributions

All authors have contributed to the conception, design, drafting, and revision of the work, provided important intellectual content; and carefully reviewed and approved the final version of the manuscript. Authors agree to be accountable for all aspects of the work, in terms of accuracy or integrity and other related aspects.

## Conflict of Interest Statement

The authors declare that the research was conducted in the absence of any commercial or financial relationships that could be construed as a potential conflict of interest.

## Abbreviations

CP/CPPS, chronic prostatitis/chronic pelvic pain syndrome; IC, interstitial cystitis; NIH-CPSI, Chronic Prostatitis Symptom Index from the National Institutes of Health; UPOINT, urinary, psychosocial, organ specificity, infection, neurologic, and tenderness system; EPS, expressed prostatic secretions; EAP, experimental autoimmune prostatitis; PAg, prostate antigens; NGF, nerve growth factor; SP, substance P; CGRP, calcitonin gene-related peptide; BDNF, brain-derived neurotrophic factor; GFAP, glial fibrillary acidic protein.
